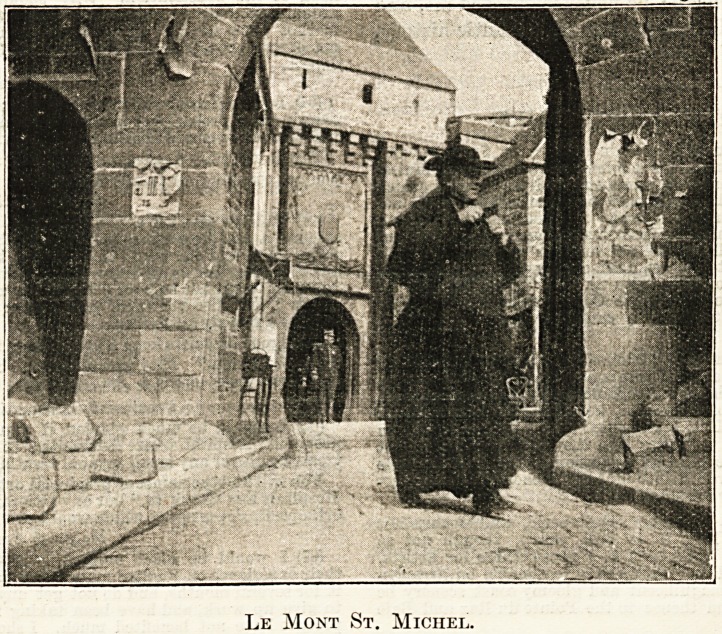# "The Hospital" Nursing Mirror

**Published:** 1899-05-13

**Authors:** 


### "The Hospital" Nursing Mirror.

Being the Nursing Section of "The Hospital."

[Contributions for this Section of "The Hospital" should be addressed to the Editor, The Hospital, 28 & 29, Southampton Street, Strand, London, W.C., and should have the word "Nursing" plainly written in left-hand top corner of the envelope.]

### Notes on News from the Nursing World.

### SERIOUS ILLNESS OF MISS FLORENCE NIGHTINGALE.

Our readers will learn with deep regret that Miss Florence Nightingale's condition is causing increasing anxiety to her numerous friends. It is well known that she has for some time been a confirmed invalid, and entirely confined to her room in the house in South Street, Park Lane, where she has resided for years. But lately her illness has, it seems, taken a more serious turn, and there is ground for apprehension that the end is not far off. We hope that it may be otherwise. Such a life as that of Florence Nightingale, whose birthday falls on Sunday, is supremely precious to the whole world. This, of course, is not the moment to attempt to appraise the value of the splendid services rendered to society in general, and to nurses in particular, by this devoted woman. But it is interesting to a generation which may not realise what the community owes to Florence Nightingale to recall the fact that 48 years have elapsed since she began her nursing work, by acting as a volunteer at the Kaiserworth hospitals on the Rhine. Miss Nightingale next nursed in Paris and London in several of the great hospitals, and made an exhaustive study of the nursing methods used in the English, Scottish, and Irish capitals. It was not, however, until she went to the Crimea that the name became a household word. There, in the hospitals at Scutari, her abilities, her courage, her patience, and her loving ministrations so endeared her to the sick and suffering soldiers that they were wont to kiss her shadow as she passed along the fever-stricken corridors. At length she herself fell a victim to the disease, and for weeks her life was despaired of. On her recovery she returned to England, the heroine of the hour, and the English people desiring to do her honour, founded for her the Nightingale Home, the pioneer of every nursing movement in existence. And yet such is the irony of events, that at the coming "International Congress of Women" Florence Nightingale, the "queen of nurses," would not, were she well enough, be eligible to address the gathering!

### MRS. CHAMBERLAIN'S APPEAL.

It is well known that the wife of the Colonial Secretary takes the deepest interest in the work of the Colonial Nursing Association, and the appeal which she has made this week for financial assistance to place it on a sound and durable basis is the more likely to meet with the response which it deserves because it has clearly been very carefully considered. As Mrs. Chamberlain reminds us, the association was formed in 1896 with a small fund raised by private subscription, with the intention of encouraging and organising the supply of trained nurses in cases where the localities had been unable to secure this provision. It selects the nurses, pays their passage out, and guarantees their salary for a term of years. If possible, the expenses are defrayed locally, and the residents in the Colonies are expected to pay for themselves as soon as the initial difficulties have been surmounted. The fact that the movement, which has the warm support of the authorities of the Colonial Office, has been thoroughly appreciated wherever the nurses employed by the association have been engaged, is an ample reason for its extension. Just now the pressing need is in our tropical possessions, and, in order to meet it, Mrs. Chamberlain asks for the sum of £5,000—not a large amount when it is remembered that the lives, as well as the health, of many of our valued public servants are at stake. No apprehensions are expressed by Mrs. Chamberlain that there will be any difficulty in the supply of nurses, if duty is offered to them. She knows that they will not shrink from the risks which every European woman must encounter in pursuing her profession under the most deadly of climatic conditions.

### HOLLAND AND WALES.

A correspondent contrasts in a striking manner the precautions which are taken against infection in Holland and Wales. Nursing a mild case of scarlet fever in Utrecht, she found that the doctor immediately reported it to the municipal authorities, and a man was sent by them who pasted a label across the front door with the equivalent in Dutch, displayed in large letters, of "Infectious Disease, Scarlet Fever." There it remained until the peeling was complete. When the doctor considered that all was safe, two inspectors first thoroughly disinfected the room and its contents, and then removed the label. Subsequently, our correspondent proceeded to nurse a case in a quaint old town in the heart of Wales, and here a very different state of things prevailed. Diphtheria had been prevalent all the winter, there were many cases of typhoid and scarlet fever, several of erysipelas, and in a fortnight five of puerperal fever. "Yet," she adds, "the schools are not closed; dangerously infected houses are not marked in any way; and the District Council are only now making a tardy inquiry concerning this alarming state of things." Most people will certainly agree with our correspondent that the Welsh people might learn something from the Dutch with advantage.

### HAMPSTEAD HOME HOSPITAL AND NURSING INSTITUTE.

The garden fête in aid of the building fund of the Hampstead Hospital has been fixed by the Princess Christian for Thursday, June 1st, and by permission of the trustees it will be held in the house and grounds of The Golder's Hill, Hampstead Heath. It is hoped that the fête will result in raising a considerable amount towards the £17,000 to £20,000 required. From the annual report of the hospital we learn that there has been no change in the method of management of the nursing staff, though a new feature in the department, intended to encourage the highest attainments in the science and art of nursing, is the presentation of a silver medal to the best qualified nurse of the service, as an enduring recognition of the work done. The first award was to Nurse Helena Stevens, to whom this notable mark of encouragement was recommended by the medical staff and by the sister superintendent. Five nurses received the certificates of proficiency in medical and surgical nursing and massage, four of whom were admitted to the private nursing staff. It is stated that several of the qualified nurses attended and appreciated the lectures in anatomy, medical, surgical, and practical nursing given to probationers. As to the private nursing staff, this has not fulfilled the expectations cherished. It has been found that the institute cannot compete with the advantages offered by the co-operative system for supplying trained nurses to private families; and "many of our nurses," says the report, "have found ready employment with them, and obtained excellent salaries and other advantages which we could not offer them."

### THE TRAINED NURSE IN THE VILLAGE.

It has been decided by the parishioners of the village of Swanwick, in Derbyshire, to have a trained nurse at a cost of £60 a-year. Dr. Parry Jones, in urging the importance of the step, contended that a trained nurse was essential in every village, and more especially so in a village inhabited by miners. Perhaps even more significant was the speech of a working man, Mr. Samuel Marsh, who also insisted upon the necessity of Swanwick possessing its own nurse. Mr. Marsh referred to the excellent services rendered by the nurse at Alfreton to the working classes, and said that they would not now like to be without one on any account. Another speaker alluded to the valuable work done by the nurse at Ripley. In the rural parts of Derbyshire the feeling is clearly gaining ground among all classes that no village large enough to have a parish council ought to be without a trained nurse.

### THE NURSES AT THE PRINCE ALFRED HOSPITAL, SYDNEY.

The annual meeting of this hospital was held on Friday, February 17th, and, at the conclusion, the Viscountess Hampden, wife of the retiring Governor, presented the prizes gained by the nurses at the examinations in the training school. They consisted of medical and practical works, which the successful nurses were allowed to select, and when the choice was made the hospital seal was stamped in gold on the outside of the cover. After the distribution tea was served in the sisters and nurses' sitting-rooms, and many of the numerous visitors availed themselves of the opportunity of visiting the wards. For the examinations 39 probationers and nurses entered, of whom 14 were probationers, 14 junior nurses, and 11 nurses. The third year first prize was obtained by Nurse Thurlow, and the third year second prize by Nurse Matchett. Nurse Hayley won the second year first prize, and Nurse Phillips the second year second prize. Of the special prizes in the second year, Nurse Hayley was awarded the prize for general ward work, Nurse Bradford and Nurse Somerville those for bedmaking, and Nurse Bradford that for bandaging. Probationer Scobie secured the first year first prize, and Probationer Page the first year second; while Probationers White and Barber received the prizes for invalid cookery.

### THE BRITISH OPHTHALMIC HOSPITAL.

The "Café concert" held at the Grafton Galleries on Monday, 8th inst., in aid of the funds of the British Ophthalmic Hospital, Jerusalem, was a very smart function indeed. The Prince himself was there and Princess Louise, as well as the Marquis of Lorne. The Duchess of Devonshire, Lady Egerton of Tatton (Duchess of Buckingham), Lady Wodehouse, Lord and Lady Knutsford, Lord Loch, and scores of other people of note in society and in politics crowded the rooms; while a whole galaxy of stars of the musical and dramatic world contributed to the programme. The rooms were lent for the occasion, and it is expected that the charity will benefit to the extent of some £300 by the entertainment.

### THE BERMONDSEY NURSES.

The sale of work last week at the Bermondsey Settlement, in aid of the nursing department, was well attended on both days, the feature on Thursday being the opening by Miss Balfour, sister of the First Lord of the Treasury. During the year which ended September 30th, the two Queen's Jubilee nurses, who undertake the district nursing of the parish of Rotherhithe, paid 5,766 visits to 203 distinct cases. Since then the staff has been increased by the appointment of a nurse whose duty it is to visit the poorer Board schools in Southwark and treat ailments of the children which, while too slight to necessitate medical assistance, still cause considerable inconvenience to the children. A grant towards the salary of this nurse is received from the Central School Board Nursing Association, but a considerable balance has to be raised locally.

### BIRMINGHAM WOMEN HEALTH INSPECTORS.

Of the four ladies who have just been appointed to serve as health inspectors under the Birmingham Health Committee one is Mrs. Nonan-Slaney, who has had experience as a nurse, and worked for some years in Liverpool in connection with Monsignor Nugent. Miss Phillips, of Stechford, has for twelve years done excellent work in one of the worst parts of Birmingham. The other applicants elected are Miss Riley and Miss Gardiner. As there were nearly 100 candidates for the four posts, the successful ones may be congratulated upon the proofs of capacity which they must have furnished to the committee. The new inspectors enter on their duties immediately, four districts in the centre of the city having been apportioned to them.

### THE VICTORIA HOSPITAL, BOURNEMOUTH.

Few provincial hospitals possess a more attractive exterior, or are better situated, than the Victoria Hospital at Bournemouth. Built of grey stone, the elevation is particularly picturesque in outline. Standing on the high ground westward of the town, breezes from land and sea keep the wards fresh and sweet, and the hospital is surrounded by a well-planted garden. The latest structural improvement is a very important one, namely, a new operating theatre, thoroughly up-to-date in construction and fittings. This addition has been built at the back of the main block. The roof is of glass, the flooring is terrazzo, and the walls are treated after a new fashion, for which the material was brought from France. With a surface resembling opaline, instead of being broken up into small squares, the walls are panelled in smooth sheets from floor to roof. The effect is excellent, and nothing could be better from the sanitary point of view. The fitments are on strictly aseptic lines, all glass and metal, and the electric light is laid on. Beneath the operating-room, which is separated by a tiled lobby from the hospital, an airing-room has been instituted. Miss Airey and her staff are naturally rejoicing in these welcome improvements.

### STORMS IN A TEA-CUP.

THE ways of guardians are oftentimes instructive, not infrequently in a sense quite unsuspected by themselves. The proceedings of the Chelsea Board afford fairly constant food for reflection to the interested observer. A number of its members seem to be far more occupied in the engaging occupation of heckling the matron, the chairman, and a few other privileged persons than in forwarding the best interests of the poor they are supposed to "guard." A question of the nurses' salaries came before the Board the other day, certain changes being proposed, which it was decided to submit to the approval of the Local Government Board. Subsequently, the chairman received information from the matron which led him to think that alterations might with advantage be made in these proposals, and he therefore took steps to hold back the letter to the Local Government Board until the question could be further considered. A simple matter, one would think, on the plain assumption that chairman and matron alike are wishful to act for the good of the institution they serve; yet it is made a topic for wholesale accusations by contentious guardians. It may be pointed out that if the Board had taken the trouble to obtain the opinion of the matron in the first instance on a point affecting her department, they would have done the more obviously proper thing; but, not having done so, at least the chairman must be conceded to have acted within his rights in giving due weight to information subsequently received.

### CENTRAL BUREAU FOR THE EMPLOYMENT OF WOMEN.

THE Central Bureau for the Employment of Women has just issued its first annual report. The main function of the bureau, the collection and dissemination of information concerning all women's professions and employments, and the giving of advice in connection therewith, is a feature of very great usefulness, for a hindrance in the way of women workers has hitherto been the almost insurmountable difficulty of obtaining really reliable information. Many women are condemned to uncongenial and unfitting work for want of the right knowledge at the right moment, or perhaps for the word in season which might have materially altered the whole tenor of their lives. For the present the committee of the bureau have to ask for subscriptions and donations to the extent of about £200 annually, since the fees paid, though increasing, do not cover the yearly outlay of £300. It is, of course, too early to say whether the undertaking will eventually become self-supporting. During the past year the following employments, amongst others, have been investigated:—Dispensers, prison matrons, telephone clerks, teachers under the School Board, matrons under the Poor Law, gardeners, and laundry matrons. The Countess of Dudley is president of the committee, Miss Margaret Bateson is hon. secretary, and Miss M. G. Spencer, secretary.

### ASSOCIATION OF NATIONAL HEALTH WORKERS.

THIS association is to be congratulated on the admirable series of lectures (organised by its hon. treasurer, Miss Ravenhill, and Miss Gray, hon. secretary) which concluded on Wednesday. If the array of well-known names on the programme aroused high expectations they have been justified in the result, for the thoughtful and well-considered addresses cannot have failed to be most helpful to the "Health Workers." Mr. White Wallis spoke on "Mental Training in Childhood"; Dr. Kenwood on "The Causes and Prevention of Summer Diarrhœa"; Mr. Loch on "Thrift"; and Sir Richard Thorne Thorne gave an extremely interesting lecture upon "The Domestic Control of Tuberculosis." Dr. Bond's address on "Vaccination" was very greatly appreciated, as was also the excellent lecture on "Food Values" by Dr. Hutchison, and Mr. Humphreys' concluding one on "Maternity from a National Point of View."

### SHORT ITEMS.

MUCH gratification is expressed at the Royal Free Hospital at the signal success achieved by Miss S. F. Fox, who was formerly a student at the London School of Medicine for Women. Miss Fox headed the list in the examination in medicine and surgery at the Durham University, and took honours in the final for the degree of Bachelor of Medicine.—Miss Weston, whose marriage is announced in our columns to-day, received her training at the East London Hospital for Children, was for a time at West London and Miller Memorial Hospitals, and for six years sister at the Devon and Exeter Hospital.—During the winter the B.W.T.A. arranged a course of physiological lectures in the North of England on the Mental and Physical Aspects of Intemperance, the lecturer being Nurse Jessie Mackay. A summer school of scientific temperance instruction will be inaugurated by the association at Barnard Castle on June 10th. The lecturers will be Miss Mary Sturge, M.D. (Bradford), Dr. Bickersteth, M.R.C.S. (Liverpool), C. L. Rothera, B.A., the Hon. Mrs. Bertrand Russel, and others.—A trained nurse, under the auspices of a representative association, has commenced her duties at Newhaven. Members are entitled by the payment of a penny a week to her services, and it is believed that they will be greatly appreciated in the town.—For three years past the nurses of the London hospitals and metropolitan nursing institutions have been invited to spend the day at Hertingfordbury. The object has been to combine with an opportunity of physical rest the spiritual help afforded by two short services held in the parish church. By the invitation of Canon Burnside this provision will be renewed on Thursday, June 1st, when the Bishop of Southwark has kindly consented to give the addresses. The only expense to the nurses will be a railway fare of 2s. 6d., and those who can be spared from their work will be warmly welcomed. All information may be obtained from the Rev. A. G. Locke, chaplain of St. George's Hospital, or Canon Burnside, rector of ertingfordbury, Hertford.

### On the Modes of Dying and the Signs of Death.

SOME time ago a useful Lecture by T. E. Hayward, M.B., F.R.C.S., was published in *Nursing Notes*, and, believing it would be of interest to our readers, we reproduce the most important portions of the article :

"In your daily work in the wards you have, all too often, the opportunity of seeing that in spite of everything which the highest medical and surgical skill and the most devoted nursing can accomplish, the patient does not recover, but dies. It may not be, therefore, without interest to you to consider the question of how and why do patients die? When you first present this question to your minds you will probably think that the modes of 'shuffling off this mortal coil' are infinite in number. This may be so when you only have regard to the apparently actual and immediate causes of death. But, on thinking a little, you may be led to say that patients die either because they have received some injury ; because they have been the victims of some disease, or they have in the inevitable order of things flickered out like a candle by the process of natural decay. But this classification does not exhaust the subject. You have to get below the surface, and consider death in the light of physiology—the science of the natural working of the body—and find out what are really the essential and vital functions on which the continuance of life depends. You will then arrive at the conclusion that all the multitudinous causes of death, whether from injury, or disease, or natural decay, really only bring about death in two ways—(1) either by failure of the circulation, (2) or by failure of respiration : that is by the stoppage of the heart, or by the cessation of breathing.

"It used to be taught that death might commence (1) at the head, that is in the brain; (2) at the heart; or (3) at the lungs. But as the functions of circulation and respiration are largely regulated by and intimately connected with the 'central nervous system,' it is found that death which 'begins at the head,' is really brought about in one of the two ways already alluded to—failure of the circulation, or failure of the respiration. In considering a little more closely the subject of death from failure of the circulation, it may be noted that this is (1) sometimes brought about suddenly, when death is said to occur by 'syncope,' or (2) at other times led up to gradually, when death is said to occur by 'asthenia,' a word which means loss of power or strength.

"The sudden failure of the circulation, or syncope, may be brought about (*a*) by diseases of the heart, either its valves being deranged in their action, or its muscular substance being in a condition of weakness or 'degeneration'; (*b*) by the sudden stoppage of the heart through the action on it of nerve influences, as in sudden death from a v olent blow on the head, or in sudden death from strong emotion, either joy or grief; (*c*) by the effects of acute hæmorrhage, the blood vessels being rapidly emptied, the heart stops because it is not filled sufficiently to enable it to contract, it has nothing to act upon ; (*d*) from 'shock' or 'collapse.' In this condition, such as may arise after blows on the abdomen, or severe injuries to other parts of the body, the veins of the abdomen become so dilated that they really are able to hold nearly all the blood in the body, and the patient dies, practically bled into his own vessels, for sufficient blood cannot reach the heart to enable it to fulfil its functions, and it comes to the same thing as if the blood had escaped outside the body.

"The other mode of death—failure of the respiration—is spoken of as death from asphyxia. You can neither properly understand this, nor the previously-described mode of dying, until you have grasped the elementary principles of physiology. But you may, perhaps, at least comprehend that failure of respiration may be brought about in two ways: (1) Either by paralysis of that part of the central nervous system which is concerned in the carrying on of the mechanical movements of respiration—'the respiratory centre'—and this may be due to or be the result of (*a*) injury ; (*b*) disease ; or (*c*) the action of poisonous substances which may be either taken in from without, or developed within the body. For instance, morphia, a poison taken from without, causes death by paralysing the respiratory centre ; and in some kidney diseases and some fevers poisonous substances are formed which have the same action. (2) Or by such conditions as prevent the access of air to the lungs. Examples of these are: (*a*) The blocking up of the air passages by a foreign body; (*b*) the growth of 'false membrane' in croup or diphtheria; (*c*) strangulation; (*d*) drowning; (*e*) suffocation in carbonic acid gas, or in the 'choke-damp' in coal mines.

"A very important point to remember in regard to asphyxia, such as occurs in drowning, is that the heart goes on beating for some minutes after all attempts at breathing have ceased, through paralysis of the 'respiratory centre'; and it is often possible to restore life, otherwise hopelessly lost, by performing 'artificial respiration'—that is, such a series of movements as have the effect of alternately drawing air into the lungs and forcing it out again. It is most highly desirable that you should all learn how to perform this—for, having this knowledge, some day the occasion may arise for using it—and it may be your happiness to be able to do without hurry and without flurry the 'right thing at the right time,' and be thus the means of saving a life which without your aid must have been sacrificed. This matter is only incidentally referred to, as the subject of artificial respiration could not be properly dealt with in the absence of actual demonstration, and this subject alone might well occupy the whole time of a lecture."

*(To be continued.)*

### Pocket Handkerchiefs in Illness.

ONLY those who have experienced an illness, and what is worse, a tedious convalescence, know the extraordinary effect illness has on handkerchiefs. Ordinarily small, limp, utterly uninteresting adjuncts, their existence being one dull round of drawer, pocket, an occasional airing, then linen bag; but wait till you are ill, who then is master of the situation? If you are hot and restless, then its usual plan is to make itself into a ball on your neck, you being too weak to remove it. In despair you murmur "Handkerchief." Up bustles nurse, and knowing from long experience that handkerchief finding is an affair of hours, gives you a fresh one, which is usually stiff. You lie on your right side, handkerchief transfers its absurd self over to the left side ; when there is urgent need for it, needless to say, it has entirely wandered off, to be felt as a hard lump later on. Scent one, put it on your pillow, and it promptly vanishes ; put it in the pocket of your sleeping-jacket, when wanted you will find yourself lying on that side, and after much fatigue will pull the pocket inside out, the handkerchief meanwhile wriggling off to get in the way of your feet; thrust one end firmly through a button-hole of your jacket, two can play at that game, so it twists and twists so that you can't get it free, and has made itself so short it will only reach your chin. A spirit of depravity comes over the ordinary harmless bit of cambric; it would be a comfort if handkerchiefs could be inoculated with some "stay-where-you-are-put" composition. In severe illness—and the mood of the handkerchief tallies with high temperature—they have been seen playing under the bed two and three together. The only way to ensure having a handkerchief is not to want one, then who so ready to be useful? Want it, and then comes the old cry, "Where's my handkerchief?"

### The Army Nursing Service Reserve.

A CHAT WITH THE SECRETARY.

The new departure on the part of the War Office is necessarily of great interest to nurses, and I therefore gladly availed myself of the opportunity courteously afforded, in response to my request, by Lieut.-Colonel Gubbins, the secretary of the Army Nursing Reserve, of calling upon him at the Medical Division of the War Office for the purpose of a chat.

"Of course," I said, by way of preface, "the main object of the formation of the Army Nursing Reserve is to organise the nurses in time of peace, so that they may be of the greatest possible use in time of war. It would be extremely interesting just now to our readers if you would supply me with the salient details respecting the conditions of service in the Army Nursing Reserve?"

"By all means. The new regulations are just being issued. Ask me any questions you please about them."

"Perhaps the most important is the matter of the needful qualifications of candidates?"

"As to the point of age, they must not be less than 25, nor more than 35 years of age, and the lowest qualification accepted is three years' preliminary training and service in a civil general hospital. Naturally, a higher qualification is the more likely to result in a successful application."

"I suppose you require some kind of testimonial from the hospital where the nurse has been trained?"

"Certainly we do. In fact, I may say that altogether half-a-dozen documents must be produced by anyone who desires to secure a position in the Army Nursing Reserve."

"That sounds rather formidable. Will you tell me what they are?"

"The first is an extract from the birth register, so that there may be no doubt that the applicant is within the prescribed age limits. Should it be found impossible to produce this, a declaration must be made before a magistrate by one of the nurse's parents or guardians as to the date of birth."

"And the next?"

"Must be a recommendation from a person of social position—not a member of the candidate's own family—to the effect that her relations are not only thoroughly respectable, but have a good standing in society."

"May I ask why the latter point is insisted upon?"

"Because the service is composed entirely of ladies. As to the third document, the medical officers under whom the nurse has served are asked to testify respecting her efficiency in medical and surgical skill. A recommendation from the matron of the civil hospital at which she has been trained certifying that she possesses the tact, temper, and ability qualifying her for appointment is also required. The nurse must sign a statement showing whether she is single, married, or a widow, and whether she is a member of a sisterhood or society. Particulars must likewise be supplied as to the place and duration of her hospital training. Lastly, she must put in a certificate from a qualified medical practitioner that she is in good health."

"Then that is absolutely all?"

"Yes, except that the ability to speak and write one or more foreign languages is held to be an additional qualification and gives preference in selection."

"Does the Princess Christian take an interest in the selection of candidates?"

"The greatest interest, and as a rule Her Royal Highness personally interviews them."

"Is the Nursing Reserve under the control of the War Office?"

"Only in time of war. In time of peace the control is vested entirely in a specially-constituted committee, of which Princess Christian is the president."

"What is the limit to the Army Nursing Reserve?"

"A hundred members, a certain number of whom may be told off by the authorities to act as superintendents."

"Would all the members see foreign service in case of war?"

"Oh, no! Generally speaking the sisters are only expected to replace, in military hospitals, those members of the Regular nursing service who are ordered abroad. Nevertheless, in cases of pressing emergency a certain proportion may be required to proceed on service at short notice."

"How do you define short service?"

"Sometimes it is only possible to give twenty-four hours' warning. But about 10 per cent. of the nurses are available at any moment."

"Do you get most of your candidates in London?"

"No; from all parts of the United Kingdom—from the hospitals and from among private nurses."

"Are the members expected to wear any special dress?"

"There is no particular uniform, but they are required at all times to wear the badge of the Army Nursing Service Reserve. It is worn on the right breast."

"What is the scale of payment?"

"When called up for duty, the nursing sisters receive pay at the rate of £40 per annum. This pay is supplemented by a further £20 a year when a nursing sister is appointed superintendent. Instead of board and laundress, there is a special allowance of 13s. a week at a home station, or of 3s. a day at a station abroad when rations in kind are not supplied. Public quarters, fuel, and light are provided. The annual clothing allowance is £4 at home, with an extra 7s. a year when serving abroad. This does not include £2 every third year for a winter cloak and £1 5s. for a summer one."

"Is there any pension attached to the service?"

"No, but on cessation of employment nursing sisters and superintendents receive a gratuity of £20. If they have served for more than one year they are also given £10 for each year after the first if the work has been at home, or of £20 for each year if it has been abroad. Fractions of a year are calculated at the same rate."

"Are there any special conditions attached to these gratuities?"

"None, except that the record of service must have been quite satisfactory, and that the cessation of employment must have been due to causes beyond the nurse's own control. If any nursing sister relinquishes her engagement for her own reasons she forfeits her right to any gratuity."

"What is the age of retirement?"

"Fifty. It is a point of interest that a great many nurses pass from the Reserve into the regular service."

"Does the committee meet often?"

"Only occasionally in time of peace, but in the event of war frequent meetings might be summoned as a matter of urgency."

### Presentations.

Last week a number of gifts were presented to Miss Frances Alice Jones, who has relinquished the post of matron of Huddersfield Infirmary, which she has held for nearly ten years, on account of ill-health. They included a beautiful gold watch and a purse of gold, subscribed by the members of the Infirmary Board and friends, the presentation of these being made by the chairman, a silver cream jug and sugar basin from the resident medical staff, a case of nurse's instruments from the nursing staff, and a dressing-case from the servants.

### The International Congress of Women Workers.

### THE NURSING SECTION.

Since we published the article on this subject in our issue of March 25th last ("Mirror," page 265) we have received several communications on the subject, and it appears we were wrongly informed upon one point stated in the article. It is not correct that no English speakers will be permitted to take part in the discussion. It has been arranged that anyone present at the meetings may send up his or her name to the President on the occasion, and will then be allowed to speak, if time permits. The fact remains that no English woman may read a paper or act as selected speaker, a rule which debars even Miss Nightingale on account of her nationality. This regulation was intended to serve as a compromise, and we are convinced that Mrs. Creighton, through some misunderstanding, was left under the impression that this so-called compromise had been accepted by the Sub-Committee of Matrons of the large English training schools, who protested against the original arrangements for the conduct of the Nursing Section of the Women's Congress. As a matter of fact, the matrons never expressed any satisfaction with an arrangement which excluded English speakers, and sent a written decision that they could not co-operate with the Congress upon such terms. Mrs. Creighton, before this decision reached her, and acting upon the supposed acquiescence of the deputation of matrons, communicated with one or two English ladies who had consented to act as selected speakers, and asked them to take part in the discussion only. One of these ladies was Mrs. Scharlieb, who, with her usual kindness and consideration, at once consented to the altered arrangement.

Mrs. Creighton, who has been put to a great deal of trouble in the matter, is very anxious that as many English matrons and nurses as possible should attend the meeting of the section, which will last for about two and a half hours. It is estimated that at least one hour will be devoted to discussion, so that twelve speakers may take part if they will consent to occupy not more than five minutes each. In fairness it ought to be stated that neither Lady Aberdeen nor Mrs. Creighton can be held responsible for the unfortunate position in which the nursing section has been placed, so far as English nurses are concerned. The whole of the difficulty has been caused by the action of the foreign representatives of the Congress, who, acting in good faith, but labouring no doubt under a serious misapprehension, have nominated certain people to represent them in various offices on the Committee of Arrangements, owing to their absence from London and inability to attend and take part in the necessary arrangements for organising this Congress there. The proceedings which have led to the misunderstanding in the nursing section are practically due to the same causes as those which first led to the troubles in the British Nurses' Association. When it is realised that the International Congress is to sit upwards of a week, that the subjects to be brought before it include everything from ostrich farming, as an outlet for women's energies, to the simplest forms of women's work, and that the nursing section is relegated to one meeting occupying altogether two and a half hours, it will be seen that under the most excellent arrangements nursing matters could not have been dealt with adequately as the Congress is at present organised, and that for practical purposes the nursing section can have no real importance or influence. In these circumstances we would advise English nurses and, indeed, all nurses to possess their souls in patience, and to regard this Congress as having little or no interest for them as a body. In due course, should occasion arise, we have little doubt that an International Congress on nursing will be efficiently organised by those who are really representative of all that is best in the nursing world.

### Typical Patients.

### THE HYPOCRITICAL PATIENT.

You find her in the second-floor-back of a dingy house in a narrow, crowded street. She receives parish relief and is attended by the parish doctor. Her demeanour towards you will differ according to your purpose in visiting her.

If you drop in for a mere gossip she will rise to the occasion and, in spite of bodily weakness, will discuss all the scandal of the neighbourhood with the keenest relish. She will backbite the doctor and the parson, and whisper shocking stories about the most respectable people in the parish.

If you call to suggest very gently that she should go into "the house" or the hospital the force of her expletives will astonish you as she opens fire upon you for your callous indifference to the "feelings of the honest poor," who have as much right as their betters to live and die in a "little 'ome of their own."

If you have something to give her she will load you with thanks, calling down the blessings of heaven upon you without stint. If you wish to talk religion she is at your service for as long an interval as you please. You have only to listen, for upon this theme she never tires of talking, and she has many rich experiences to relate. She will give you her views at any length upon the "second coming" and the "millenium," and she will discourse freely upon "original sin" and the "sweet doctrines of Grace." She complains of the language which reaches her ear from the streets, and of the numerous signs which testify to the godlessness and depravity of her neighbours. She always takes occasion from these lamentations concerning the reprobate condition of others to tell you of her own unceasing efforts and prayers for their conversion.

The curate's visit is to her as good as a feast. Her face, usually long and dismal, broadens into a bland smile as she welcomes him. Cant religious phrases are drawled forth into his ear. Her pious speech is plentifully interlarded with sighs and groans. She tells him that she has "mostly gone to chapel herself," but that she always "liked the Church," and "bless you, sir!" she adds, "what does it matter? Ain't we all aiming at the same place? And ain't we all yearning for the salvation of poor perishing sinners?" She knows his step, and whenever he ascends the stairs she spreads her Bible open before her. Engrossed in the "precious word" she fails to notice that the sacred page is often upside down.

The woman whom he pays to look after her complains bitterly of the violent temper of her patient, and is with difficulty persuaded to stay. On approaching her door he sometimes hears her voice raised in the loud utterance of language more vigorous than elegant; but as he sits at her bedside he thinks his ears must have deceived him, so dulcet-like are her tones in describing to him the rapturous effect of the "sweet passage" she has just been reading.

The district nurse looks in now and then and is greeted with an unctuous smile, but no sooner has she left the room than she is denounced as a "presumin', meddlin', interfering thing."

When the lady visitor from the church appears the patient is seized with a violent fit of coughing, accompanied by dreadful rheumatic pains. This serves the purpose of evoking the practical sympathy of the lady, while it affords an opportunity to the sufferer of modestly professing her Christian patience and resignation.

While this visitor is with her the voices of children in the street ascend to her chamber. This moves her to speak tenderly and with a benignant smile of the "dear little angels who play together so nicely," but directly the lady is gone she orders her attendant, in wrathful tones, to drive away "those yelling brats, who torment her to death."

### Across the Seas.

### THE PROVINCIAL HOSPITAL AT PORT ELIZABETH.

Port Elizabeth is said to be one of the most "English towns" in South Africa. It is prettily situated in Algoa Bay, and the view from the Provincial Hospital, which is on one of the highest hills in the town, is very charming. The wide, shallow bay stretches out dotted with its boats and ships, smacks and small craft. When the big steamers "come in" there is always a pleasurable commotion in the hospital, since the officers of the chief liners are most generous in their hospitality to the nursing staff of the "Provincial." While the liners are in port, boatloads of nurses "put out" to the steamers to enjoy the social dissipations of cheerful luncheon and tea parties—the excursion including a nice little sea trip, since the large steamers anchor at some distance from the jetty. It is quite a feat in hill climbing to return to the hospital from a jaunt in the town. Part of the hill is so steep that steps have been cut out and a handrail provided to help the hospital visitor.

A beautiful nurses' home has been built out of funds raised in honour of the Jubilee. The cottage where the nurses were housed is now converted into a maternity hospital. The staff consists of a matron and seventeen nurses, a resident doctor, secretary, and dispenser. In addition there are two ward boys, and an orderly for the chronic ward. Add to these a heterogeneous household of Kaffir, Malay, and half breed wardmaids to complete the staff. There is provision for 120 beds, so that the Provincial Hospital possesses material for an excellent colonial training school. Enclosed in its own grounds, nicely planted with trees, the hospital is quite a picturesque feature of Port Elizabeth. Otherwise the town, which is a commercial port with large warehouses, stores, a jam and candle factory, is not delightful.

The native ward is the most modern and up-to-date ward in the hospital. This is somewhat of a waste of good material, since the Kaffir intensely dislikes ventilation and has no eye for any decorative effects which may surround him.

The sick Kaffir is not an easy person to manage. To him it is a trifling circumstance to strike or bite his nurse when she attempts to administer a dose of medicine or otherwise to enforce discipline to which he objects. Since there is no restriction in Port Elizabeth, as in many other districts of South Africa, on the sale to natives of intoxicants, a large percentage of the patients are alcoholic. Kaffirs are allowed to make beer from mealies boiled and fermented, and they drink gallons of Cape brandy, which is abnormally strong. Therefore, accidents and the injuries resulting from alcoholic assault and battery are extremely common in the "Provincial."

In the prohibition districts, as in Rhodesia, such cases are extremely rare; and in Cape Colony the native is well looked after and kept within bounds. As a general rule, Kaffirs suffering medically do very badly, but they make admirable surgical cases, and terribly contused wounds and scalp injuries recover marvellously, generally healing by first intention. Part of this is ascribed to the dry, rarefied air of South Africa.

The Kaffir has an intimate knowledge of native poisonous herbs, and does not hesitate to try these on obstreperous enemies, a process which is known to the Kaffir as the "removal of difficulty." So that a native ward often contains a case or two of attempted "removal" by poison. The entire hospital is lighted by the incandescent system, and the children's ward, with adjoining rooms for private patients, is really beautiful.

Natives and Europeans alike are received in this ward, since it is impossible to extend to children the many divisions of colour which must necessarily exist in a mixed racial population. The Kaffir children are quaint little persons, some of them charming, good-humoured, and very winsome in their ways. Their broken English is most amusing, and their joy after fractures at being allowed "to walk on their two legs," as they call it, when crutches are discarded, is very frank and unconcealed.

Native English is learned mostly from somewhat uneducated "white people," so that when a little Kaffir, reproved for behaving badly, seeks to shift the blame on to a black-eyed, dark-skinned neighbour in a cot near by, he probably says: "It's 'im, nurse, wot's naughty," the incident reminding one curiously of London. The women Kaffirs are mostly quiet and well-behaved; it is only the men whose habits and customs are so uncouth and repulsive.

Formerly, the native male ward was nursed by ward boys and orderlies, and the patients behaved much better then than now, when it is nursed entirely by women nurses. A project for returning to the old system is being discussed, since so many of the women nurses very strenuously object to nursing the male Kaffir without the assistance, day or night, of an orderly. The hours on duty for the nurses are long and the work is extremely hard. Food is very good, as it commonly is in South African hospitals. Under the old *régime* the wards were in the charge of certificated nurses, and all the cleaning, dusting, and sweeping was done by native servants. Since the hospital became a training school much of the domestic work has fallen to the share of nurses and probationers.

The brass knobs and gas brackets of some South African hospitals appal the average English nurse and probationer when she learns that it is her lot to polish them to spotless point. The Salvation Army some time since presented, in all generosity, a brass cot to the children's ward of the "Provincial." It forms a most decorative object in the ward, but to keep it bright it should almost be endowed with a special probationer in perpetuity. It is a white elephant, and absorbs as much time and nursing service as a "'trachy' tent." The chronic ward is somewhat curious, since it is not necessarily for sick persons. Disabled and old men who cannot work and have no place of refuge remain for years in this comfortable, well-furnished ward. Hospitals are the work of the present generation. The building and provision of almshouses will be the work of a later one.

When an outbreak of small-pox—confined, however, to a few cases—temporarily closed the hospital to all but the most urgent cases, it necessitated the quarantining of the staff. The epidemic started in Johannesburg and rapidly spread, since Kaffirs prove very susceptible to this disease. To console the nurses for the isolation and loss of outside social life during the quarantine following on three or four cases occurring in the wards, one hour's dance every evening was inaugurated. These were strictly Dorcas dances.

Private nurses do extremely well in Port Elizabeth, although it is important for English nurses desirous of emigrating to South Africa to remember that the cost of living, and the expenses of clothing and laundry, considerably discount their earnings. The fees paid to private nurses in South Africa sound to English nurses as if the country were an Eldorado of prosperity. But those who are here find that the rate of living is so high that it leaves barely half the saving margin in salaries which fancy painted. At the same time, English nurses thoroughly qualified, content to take the rough and the smooth together, and prepared to work harder than at home, can do very well throughout South Africa in private nursing.

### Echoes from the Outside World.

### AN OPEN LETTER TO A HOSPITAL NURSE.

THE Queen, who seems to have come back from Cimiez with a fresh stock of health and energy, is evidently anxious to use some of it in taking as prominent a part as possible in the birthday festivities which are now being planned. It is no trifling exertion for an old lady of eighty to drive some distance through the streets and bow continually to her delighted subjects, and it means a great deal of self denial and kindly thought, for which, I am sure, we are all grateful. Such of you as have any time off just then will have two or three chances of seeing her. As at present arranged, Her Majesty will arrive in London at twelve o'clock on Monday week, and proceed by way of London Street, Hyde Park, and Hyde Park Corner through the arch at the top of Constitution Hill to the garden entrance of Buckingham Palace. In the afternoon she will drive out in an open carriage, with equerries in attendance. Where to is not yet specified, but probably only the Park. On Tuesday the Drawing-room will be held, and afterwards the Queen will take another drive, according to her usual habit. On Wednesday there will be a thanksgiving at the Chapel Royal, St. James's, at one p.m., when the musical portion of the service will be most elaborate. Many of the Royal Family will be present, and the chapel will be decorated with great gold alms dishes and a profusion of flowers. This, of course, will only be for the select few, but for the many who will like to express to God their gratitude for the numerous blessings of this lengthy reign there will be a thanksgiving service of a more general character at St. Paul's Cathedral at eleven o'clock, when the Archbishop of Canterbury will preach and the Lord Mayor and Sheriffs attend in state. Later in the day her Majesty will drive in semi-state from Buckingham Palace to the South Kensington Museum. Take my advice and secure standing room in the Brompton Road if you can. The pavement there is high, raised a good deal from the road, and you should get a good view. The ceremony will be late in the afternoon. Nearly all the "Royalties" will be in town and attend this function, and possibly the Empress Frederick may come over for the occasion. All who can should get a glimpse, too, of Kensington Palace. On the Queen's birthday proper, May 24th, the whole of the historical place is to be thrown open to the public, and it will be possible to see the room where Queen Victoria was born, the room where she played and learnt lessons, and the antechamber where the announcement that "The King is dead, Long live the Queen!" was made to her in the early hours of that spring morning sixty-two years ago.

---

Do you know that the Russian Government has had to appoint a special commission to reform their calendar, the Julian, which is twelve days behind that used in nearly all other countries, the Gregorian? You can well imagine how awkward it would be for a nurse from England to promise to attend a case in Russia, say on June 1st, and not to know if she was due on May 20th or 12 days later. Yet, till matters are put straight in Russia, such mistakes must be of frequent occurrence.

---

I SEE that a Presbyterian missionary, the Rev. Dr. Kellogg, has been killed in the Himalaya Mountains. He was apparently riding close to a precipice, and, in some way not yet known, he lost his balance and fell over. It is quite possible that a bicycle is an exceedingly useful machine for a clergyman who has a large amount of ground to get over, even in such an apparently unsuitable locality, and that the poor man met his death in the discharge of his duty. But when I was reading the account I could not help being forcibly reminded of the fashionable pastime of the visitors at some of the Alpine hotels last year. A cousin of mine who was staying there sent me blood-curdling descriptions of the reckless way in which both men and women used to ride down steep mountain paths where there was only room for one machine to go at a time, and where a sudden contact with a stone or a "skid" of the wheel might result in certain death, for on one side lay a precipice of many hundred feet, with a rushing torrent at the bottom. Upon a memorable occasion my cousin turned the corner of a rock rather quickly, and found herself almost face to face with a man riding towards her. Fortunately the man jumped off just in time, and so prevented a disastrous collision, but in thus securing the lady's safety he lost his own machine, which went over with a sickening crash. No remonstrances had any effect in getting these foolhardy riders to relinquish their hazardous sport. "Give up mountain riding? Not exactly! There is nothing to equal the rush through the lovely invigorating air, and it's all right if you are careful." And so without a thought of the future, death was recklessly courted for a mere whim.

---

INTO the hearts of at least nineteen out of twenty women the word "burglar" strikes terror. I don't mean in the daylight, seated in a drawing-room with a choice little circle of friends partaking of afternoon tea. It is so easy to be brave under those circumstances. But wait till the shades of night have gathered in, when the wind is howling dismally, shaking each casement with an ominous rattle; when the whole house, with the exception of yourself, is, or seems to be, asleep; when every door creaks strangely, and the boards re-echo the same sound; then where is your boasted courage? I fear that what little may be left will ooze slowly out of the end of your finger-tips when you call to mind a little fact which has just been officially announced, namely, that burglars are on the increase. There is no doubt about it. The annual average of convictions for "burgling" has risen from 1,465 in the four years from 1883 to 1887 to 1,684 in 1893 to 1897, and there is no consolation to be found in the idea that perhaps the difference in the figures is due to the circumstance that the authorities are more wide awake, and catch the thieves more frequently. Not a bit of it. The police have also to report a far greater increase in the number of crimes of this class where no captures are made. I tried myself to derive joy and comfort from the statement that the City Police Force had been increased by 73 constables. But it was no good. The extra men, I find, are to look after the street traffic, not our household goods. So there is nothing left for me but to hope that my turn will be long in coming, and for you to congratulate yourself that you live in a hospital, which is not generally looked upon as a happy hunting ground for the purloiners of property.

---

FOR the want of a trifling caution we all of us frequently run a serious risk of injury without pausing to think about it. I daresay nurses are just as bad as other folks, but perhaps I am maligning you, and it is only foolish people like myself who are so thoughtless. I refer particularly to not scoring the soles of new boots before putting them on, and thus removing the tendency of the wearer to slip. I seldom remember to take the precaution, and yet I could call to mind half-a-dozen accidents in my own personal knowledge from this cause. As recently as Wednesday a man carrying coals at Edinburgh slipped down the stairs and injured himself so badly that he died. He was wearing new boots with slippery soles.

### The Nurses' Bookshelf.

[We invite Correspondence, Criticism, Enquiries, and Notes on Books likely to interest Women and Nurses. Address, Editor, The Hospital (Nurses' Book World), 28 & 29, Southampton Street, Strand, London, W.C.]

Burdett's Official Nursing Directory, 1899. A Directory of Nurses, compiled and edited with the assistance of a small committee of medical men and matrons, by Sir Henry Burdett, K.C.B. (London: The Scientific Press, Limited, Southampton Street, Strand.) 5s.

We are glad to receive the new issue of the "Official Nursing Directory." The organisation which was sufficient to bring out the first annual volume after considerable time and labour, has been more than sufficient to keep the present number up to date and to improve its usefulness in many respects. Speaking from our own experience the directory has been of much service; we have been able to ascertain the addresses of nurses on more than one occasion when we wished to obtain them on an emergency for employment; and from the point of view of the nurses, this must always be one of the main uses of the directory. We are glad, therefore, to see that 1,100 names of nurses have been added to the directory this year, and we hope that in time the directory will become complete, and, therefore, of still greater service to the medical practitioner. The other portion of the volume which has been of service and interest is the part devoted to the description of the hospitals and infirmaries which train nurses. No doctor, especially no hospital physician, passes much time without having requests from the female relatives of friends who wish to commence nursing training. The rush of women to learn nursing seems still to be on the increase. The information put at our disposal by the official directory has been of much service in guiding us in this respect. We are sorry to observe that there are still one or two prominent exceptions to the rule that all the training schools give complete information to the directory. In our own experience this want of detail has given unnecessary trouble in correspondence. It appears to be unfair to blame the Editorial Committee for this want of information, for we observe that the editor keeps strictly to the meaning in which he uses the word "official." As applied to this directory it signifies "that the information given in it related to nurses in training schools is either derived from official sources or has been verified by competent authorities." It is clear that information of any other sort might be of more trouble than good service. The directory has come to stay, and, speaking from the point of view of a medical practitioner, the more complete the information both as regards the nurses and the training schools the more likely are nurses and schools to benefit. We notice that a small section is devoted to Colonial, American, and foreign hospitals and nursing institutions. These institutions certainly do not suffer by comparison with those at home. Sir Henry Burdett and the Editorial Committee are to be congratulated on the completion of their second year's labours, and we trust that the work in future will be less arduous and the result still more satisfactory.

### Death in Our Ranks.

We regret to have to record the death of Miss F. Cox, night superintendent of the Middlesex Hospital, which took place on Saturday last after a short illness. Miss Cox was a probationer at the Middlesex Hospital from 1875 to 1876. She returned to the hospital in December, 1878, as night superintendent, which post she held up to the time of her death. Miss Cox will be much missed at the hospital, where she was respected by all who knew her for her high personal character and conscientious performance of duty.

### Novelties for Nurses.

### SPRING APPAREL.

Many and varied are the attractions offered to customers at this popular establishment, and never more so than at the present time. Leaving, however, the bright-hued silks, delicately embroidered muslins, and dainty lingeries on one side, with stern resolution we pass on to the equally interesting articles with which our readers are more directly concerned. These have their own special points of attractiveness, and at no establishment is the useful and the beautiful more successfully blended than at Messrs. Garrould's. There are several new designs in cloaks, especially the "Cavendish" and the "Cecil," both of which are eminently up to date, but while admiring them we still retain a strong predilection for the time-honoured "Angelus," which is the most desirable because the most suitable of all. The "Wellesley" is a new form of dress suitable for cycling, and very convenient. The skirt fastens round the waist and buttons down the front, and a loose-sleeved jacket three-quarter length is worn with it. In waterproof serge cloth the price is 35s. 6d., and it is of feather-weight. In caps there are some charming designs, the "Abbeville" being a particularly becoming shape, and they all have the merit of going flat for washing when the string is untied. Cuffs and collars are in great variety and also linen sleeves suitable for operation purposes. The sick-room slipper and silent ward shoe still retain their popularity, and we are promised fresh novelties in this department before long, which will merit a special notice for the benefit of our readers. In dress material the choice is enormous, and we admired the Harisburg beige as being one of the best materials we have seen as yet for nurses' wear. Private nurses could not do better than have a dress of this material for wear in cold weather, or when sitting up at night. It is much warmer than cotton and washes equally well. In colour it is a soft grey and could be worn alternately if desired with grey zephyr. We are glad to see that Messrs. Garrould have brought out a new aseptic wallet, which is so constructed that it is impossible for dust to become lodged in any part of it. In real morocco the price is 6s. 6d., and very good value it is for the money. The folding chart board is an excellent contrivance by which the interior is kept clean and tidy. A conveniently-shaped bath thermometer will be found a decided improvement on the clumsy old-fashioned kind with which we are so familiar, and we feel assured that the heart of all district nurses will open towards the really admirable bag which has been so carefully fitted up for their inspection and approval. Medical books can also be purchased on the premises, and nurses are offered gratis the use of the London, Nursing, and Medical Directories should they require them—a boon which has only to be known to be appreciated. Last, but not least, after a busy day's shopping we must refer our readers to the well-appointed tea-room, where all kinds of light refreshments are obtainable at a moderate cost.

### THE BEST PERFUMES.

We have before us samples of the celebrated 4711 Eau and of the delicious perfume "Rhine Violets," distilled by Messrs. Mühlen, of Cologne, and to be procured at 62, New Bond Street. Both these specialities are quite unrivalled, and form a delightful addition to the sick room, where only the very best perfumes should ever be introduced.

### Resignations.

Miss Sprague, matron of the Spalding Johnson Hospital for the last fifteen years and a half, has resigned her post. The resignation, which the trustees accepted with much reluctance, will not take effect until July. Miss Spragge, who joined the Nightingale School of St. Thomas's Hospital in January, 1870, is giving up active work.

### A Book and its Story.

A NEW NOVEL BY M. E. FRANCIS.

MRS. FRANCIS BLUNDELL'S story of "Miss Erin"* is delightful. Full to the brim with racy character sketches, teeming with the national traits inseparable from delineation of Irish character, there is one unpleasing exception to the generous, impetuous persons to whom we are introduced in the character of Fitzgerald of Glenmore, whose harsh and parsimonious disposition adapts itself admirably to the part of the wicked uncle, which he plays throughout the early part of the story.

In '48 Gerald Fitzgerald, his younger brother, was charged and convicted of treason-felony. Some years later he, with the leaders of the movement, received his pardon, and at once made his way to America, where, after a wandering, unsuccessful life, he settled down on a ranch in the Western States, taking for his working partner a countryman, Michael Dooley. There he met a pretty Irish peasant girl recently arrived in the district. Something in her blue eyes and forlorn position touched his too susceptible heart, and, although no longer a young man, he married her. At this time affairs on the ranch were in a serious condition, and Michael had vainly endeavoured to impress upon his optimistic partner the necessity of giving it up if they were not to come to utter ruin. Perhaps the disease to which he ultimately succumbed caused this lethargy and indifference to affairs, for it was only after further energetic protests on Michael's part that he consented to part with the ranch. After two years of married life, which, in spite of the difference in social position, were happy ones, Fitzgerald's wife died, leaving him a little girl of a few weeks old. He was not long in following her, and the tiny "Erin," named by her father after the land he loved so dearly, was consigned to the care of the faithful Michael for safe delivery into the hands of her uncle in Ireland.

It was a bleak autumnal evening when the oddly-assorted pair arrived at the inhospitable doors of Glenmore, and poor Michael's appearance, carrying the precious burden in his arms, tenderly borne through the dangers and discomforts of the long journey, was doubtless not reassuring to the elderly servant Martha, who opened the door to him. Visitors there were rare, for the master of Glenmore neither sought society nor welcomed visitors to his cheerless home. In the waning light Michael is taken for "a wandering vagabond," a not unnatural assumption, favoured by his fierce, unkempt appearance.

"Go away," she cried sharply, "we've nothing for you. We don't encourage tramps here." Michael, naturally indignant at her address, and feeling the importance of his mission, replies, "Keep a civil tongue in your head, if you plaze, an' tell your master that Michael Dooley, MISTHER Michael Dooley, of Lincoln Creek, California, United States of America, 'ud be glad of a word with him; an' I guess you'd best tell him I've got something for him."

Martha responds by shutting the door in Michael's face. She had gone in search of her master, who appears shortly and interrogates his strange visitor through the half-open door. The replies he received did not serve as a salve to his suspicions.

"What have ye brought me?" asked Fitzgerald.

"I've got a letther for ye from your brother in California that he wrote when he was dying. It's about a small leggicy he's left ye."

The news of his brother's death, an event which he imagined had taken place long ago, did not visibly affect Fitzgerald. "So Gerald is dead," said the old man with perfect unconcern. "And he left me a legacy, did he? It won't be much, I should think, coming from that quarter. However, we'll see; hand over the letter."

Michael declines to give up the letter, as he had promised to see it opened and read in his presence. In the gathering darkness this is impossible, so, pushing by Fitzgerald, he makes his way into the narrow hall, and from thence to the dismal room which formed Fitzgerald's sanctum. The face which bent over the unfolded paper was a cruel face, too, yet with a certain remnant of beauty in it, even a faint resemblance to the poor dead brother who had been the traveller's friend. In the silence of the room, broken only by the weird moaning of the wind through leafless boughs without, Fitzgerald perused the letter, and then, turning abruptly to Michael, asks for an explanation. Unfolding carefully the wraps in which his little charge is enveloped, he shows the face of the sleeping child.

"This is your leggicy, sir. The only child of your dead brother. Its mother died just after it was born, an' poor Mr. Gerald himself, I may say, only lived long enough to see it christened and give it his blessing, and bid me take it to you." The unconscious cause of these remarks slept placidly under the withering, malignant glance of her newly-discovered relative, who declines to believe without more convincing proof that she is his brother's child. Assertions and protestations alike cannot avail to make him accept the guardianship of the child. "There, take the brat away. I'll have nothing to do with it."

The solution to the difficulty which Fitzgerald offers, that of sending the child to the workhouse, is one which Michael indignantly rejected. And yet how can he, "a bachelor," be burdened with an infant?—so, gently folding the wraps around the sleeping child, he trudges out into the night once more, and falls in, a few paces further, with the kindly humorous priest, Father Lalor. As briefly as an Irishman can, under such exciting circumstances, he explains the situation, and its connexion with Gerald Fitzgerald. The name recalls kindly memories. "Gerald Fitzgerald! Yes, indeed I knew him. A fine lad, a fine, brave, generous, foolish lad. And so he is dead! May God have mercy on his soul."

Through the kindly offices of the good priest a home is found for the little Erin in a peasant's family, and Father Lalor takes her under his fatherly eye, supplementing the miserable dole which has been unwillingly granted by her uncle with many generous gifts of his own.

The record of "Miss Erin's" childhood is a varied and unhappy one, arousing all the latent defiance against tyranny and injustice which was characteristic of a temperament inherited from her father. Sharing with him a proud, passionate nature and an unyielding, if sometimes mistaken, sense of justice, she grows up with the same desire to ameliorate the wrongs of her beloved countrymen by which he was moved. As time goes on, and she becomes possessed of the large fortune to which, upon the death of her uncle without a will, she finds herself sole heiress, she is able to put her desire to practical use. She suffers severely upon one occasion for her mistaken zeal; and, belligerent little person as she is, even fights her lover to the bitter end, which comes to a sweet conclusion after all, in spite of past resistance.

From the first to the last page the interest of the story never flags. In her simple yet powerful character sketches, Mrs. Blundell recalls the Brontës, and there is a freshness and sincerity in the portrayal which is infinitely refreshing.

* "Miss Erin." By M. E. Francis (Mrs. Francis Blundell). (London: Methuen and Co., Publishers. 6s.)

### OUR CONVALESCENT FUND.

WE beg to acknowledge with many thanks a donation of 2s. to the above Fund from Nurse E. Parker.

### Everybody's Opinion.

[Correspondence on all subjects is invited, but we cannot in any way be responsible for the opinions expressed by our correspondents. No communication can be entertained if the name and address of the correspondent is not given, as a guarantee of good faith but not necessarily for publication, or unless one side of the paper only is written on.]

### A DEARTH OF PRIVATE NURSES.

"Iris," writing on the same question, says: I suggest that all nurses who are not contented with private nursing institutions should enter one like Guy's Nursing Institution, where all the nurses are well looked after, well fed, and very kindly treated. If in for the night they never need appear at breakfast till a quarter to nine, and can indulge in late hours in bed in the morning or early hours at night by asking permission. They are allowed to go out and come in when they like if it is possible, of course getting sanction from the lady superintendent. The salary, too, is all that could be desired, rising gradually to £40 a year. A part, and a large part too, of every nurse's pension is paid by the institute. In fact, the institute is a really happy home, and the nurses look forward with pleasure to return to it after each case. It may, perhaps, be as well to add that a three years' first-class Guy's Hospital certificate is essential before entering on the private staff.

"N. F." writes: I think my case will furnish one explanation of the dearth of private nurses. I have been a nurse for the last ten and a half years; I was trained (eighteen months, a much longer period than many probationers at that time) at a large provincial hospital, and worked there in the wards and on the private staff for eight years, when for private reasons I sent in my resignation. I left with a certificate for the eight years' work, and the best of testimonials from doctors and matrons, which any woman might be proud of. Later I trained at one of the principal lying-in hospitals in London, gained my certificate for monthly nursing, and afterwards inquired my best way to join one of the private nursing institutions. At the Nurses' Co-operation I was informed I could not join without a three years' certificate. At the same time I knew perfectly well that one or two of the nurses who first joined it were trained with me, and are still on their staff. I inquired at others and received the same reply, and so preferred taking up work on my own account, which I am glad to say has up to the present answered very well.

Nurse B. writes: I am very pleased that the question has been asked as to the cause of nurses objecting to join the old-established nursing institutions. One of the strongest reasons is, I am sure, that a nurse loses her individuality and becomes simply a part of the machinery. She is sent out to a case, and if she is conscientious she does her very utmost to to assist the medical adviser to bring about her patient's recovery. Then, if the case has lasted some weeks she goes back to the institute with a big cheque—not for herself, she gets very little out of it—and is sent out to the next case for which the superintendent thinks her fitted. So her life goes on, week in, week out, knowing that very little interest is taken in the home as to whether she is over-tired, or in need of a few encouraging or sympathetic words, or a little pleasure or recreation. Then, as to life in the home between cases, especially where "sisters" and "nurses" are kept in the same home. The "nurse" is not allowed to join in the conversation at the table unless addressed by the superintendent, nor to go out for a walk until she has asked permission, and there is also a general feeling of constraint all through the day, so that she is only too glad to get away to another case, where she is at least treated with courtesy, and oftentimes with great kindness. The salary varies from £30 to £34 per annum, for which she has to work a whole year, with just one month's holiday, and this, added to several annoyances of a minor character, causes her, when she sees a way out of it, to immediately fly to the remedy, viz., joining a co-operative institute. Then she receives her own fees, paying at the rate—at the most—2s. in the pound commission, and finding her own room and food when not at a case, and being absolutely free in the matter of religion, recreation, &c. I would advise any nurse either to work on her own account or to join a co-operative institute in preference to being part of the working machinery of a private nursing institution. Of course, in the latter case the salary is sure whether the nurse is working or not, and the institute promises medical attendance when ill, but frequently one has to go into lodgings when getting convalescent, often a matter of 30s. a week. This soon takes the gloss off £30 a year salary, especially if the work has been pretty hard through the winter, so that getting well is a long job. I have tried both, and I would much rather be on the co-operative principle than in a private nursing institute. These institutions, however, might become a success if the matron would let her nurses feel that they are individuals and members of the home family, and not merely cogs in the wheels of the machinery. If nurses knew that an interest was taken in them and their work, an encouraging word given them on returning from a case, and as much rest as possible granted them between whiles, in short, that the matron was their *friend*, then the nurses would stop, and perhaps induce their acquaintances to join them.

### THE POSITION OF NURSE IN A POOR LAW INFIRMARY.

"A Lover of the Profession" writes: I have read with very much interest the numerous comments on Poor Law infirmary nurses and their position. Being a superintendent myself, and one of the less fortunate ones, I do feel something ought to be done to raise the standard of the various institutions and to help to make the nursing staff more comfortable. I heartily congratulate the superintendent who has been fortunate enough to get with such a nice matron; but in nine cases out of ten the position is anomalous. I had a letter from a superintendent not long ago in which she said, "I do wish Local Government Board would make our position more definite." This, I fear, is the cry of numbers of nurses holding the position of superintendent, and I feel sure most, if not all, would be only too glad if the petition which has already been suggested could be signed and sent up. It is an excellent suggestion. I think we should not forget Miss Wilkie, as well as Miss Wood, in our thanks, and I wish their efforts met with greater success.

### "ARE NURSES EXTRAVAGANT?"

"Nurse B." writes: I am sorry to contradict "A Nurse of Twelve Years' Experience," but I would say, as a rule, certainly, No; how can they possibly be? Although not on the co-operative principle, I am a privatenurse, and on behalf of myself and others I should like to say that those private nurses who are so dreadfully extravagant are much more fortunate than we have been if they can indulge in "whatever pleases their fancy" from the large fees which they are supposed to get. I am sure I speak for many when I add that so numerous are our rests "between cases" it takes us all our time to get necessaries, much less to indulge in luxuries. A nurse friend of mine said to me recently, "Three cases per annum is not very lucrative business now, is it?" I should think not. Certainly neither of us have built up a "large connection" as yet, but the doctors so kindly keep on saying they will remember us, and I have no doubt they will some day. Consider what private nurses have to do with their money. They have to keep themselves "between cases," and as too often happens, they occasionally get a very lengthy rest. Then the money flies very rapidly, but not in theatre-going, hansoms, &c. Surely these so-called extravagant, reckless, careless, pleasure-seeking, spendthrift, betting nurses must be few and far between. Speaking from my own and my friend's experience, nurses learn how to stint and pinch in an incredibly short space of time. A lady in London recently told me that good nurses can be had for 10s. per week. Can this be true? If so there is not much chance of over-indulgence in any form of extravagant pleasure. I love nursing for its own sake, and it is well that I do, for the "cash in hand" from nursing never seems to attain a large sum.

### Appointments.

BRISTOL JUBILEE CONVALESCENT HOME.—Miss Annie Ellis has been appointed Matron. She has been night superintendent at the Bristol General Hospital since 1894. She was trained at the Royal Portsmouth Hospital, and her appointments have been charge nurse at Portsmouth, private nurse at the Tunbridge Wells Institute, sister in surgical wards at Queen's Hospital, Birmingham, and charge nurse of fever wards at the South-Eastern Hospital, London.

VICTORIA HOSPITAL FOR CHILDREN, CHELSEA.—Miss Jane Watson has been elected Matron. She was trained at the General Infirmary, Leeds, and her appointments have been infirmary matron of Portsmouth Hospital, home sister of Victoria Hospital for Children, Chelsea, and matron of the Victoria Hospital for Children, Hull.

VICTORIA PARK HOSPITAL.—Miss Amy L. Burleigh has been appointed Assistant Matron. She was trained at St. Bartholomew's Hospital, and has been night sister at Victoria Park Hospital since September, 1898.

HOSPITAL FOR CONSUMPTION, BROMPTON.—On May 4th Mrs. Ellen Price was appointed Lady Superintendent. She was trained at Guy's, and her last post was at the Mill Road Infirmary, Liverpool.

### Minor Appointments.

BIRMINGHAM EAR AND THROAT HOSPITAL.—On May 3rd Miss Gertrude Holmes was appointed Sister. She received four years' training at Queen's Hospital, Birmingham.

VICTORIA PARK HOSPITAL.—Miss A. Richardson has been appointed Night Sister. She was trained at the Bolton Hospital, and has held the post of charge nurse since August, 1898, at Victoria Park.

CORRECTION.—Miss Annie Wood, whose appointment to the post of Head Nurse at Camberwell Infirmary was announced last week, desires it to be stated that she was trained at the Salford Royal Hospital, Manchester.

### Where to Go.

ROYAL BRITISH NURSES' ASSOCIATION.—The Café Chantant in aid of this association will be held at the Hotel Cecil on May 15th, under the presidency of the Princess Christian. The list of patronesses is a most distinguished one, and the Prince of Wales has promised to be present. The entertainments, under the management of Sir Henry Irving and Mr. George Alexander, should be most attractive, as most of the leading actors and actresses are giving their services. Tickets, one guinea each, may be obtained from the box office of the St. James's Theatre, or from any of the Lady Patronesses.

FREE HOME FOR THE DYING, 82, The Chase, Clapham, S.W.—A concert and variety entertainment in aid of the above will be given at Grosvenor House, on Tuesday, May 16th, 1899, at three p.m. punctually. Mrs. Mary Davies, Madame Clara Samuell, Miss Marion Arkwright, Mr. Nicholl, Mr. W. G. Elliot, Mr. Rutland Barrington, Mr. J. S. Liddle, and Mr. Cyril Maude, have kindly given their services. Tickets, 10s. 6d. each, may be obtained of the secretary, 82, The Chase, Clapham, S.W.

RECITALS.—St. James's Hall: Herr Ludwig Strakosh and Mdlle. Marie Boedcher will give a vocal recital on Monday, May 15th, at three p.m. Queen's Hall, Langham Place: Miss Mary Owen will give her second and last recital on May 18th, at three p.m.

CONCERTS.—St. James's Hall: Madame Edith Grey-Burnand will give a grand evening concert on Wednesday evening, May 17th, at eight p.m.; Mdlle. Anna Kuznitzky, Mdlle. Marie Heimlicher, and Mons. Aurel v. Belinski will give a vocal and instrumental recital on Friday, May 19th, at hree p.m.

### For Reading to the Sick.

YOUR heart shall rejoice, and your joy no man taketh from you.—*St. John* xvi. 22.

Rejoice in the Lord alway; and again, I say, rejoice, and the peace of God, which passeth all understanding, shall keep your hearts through Christ Jesus.—*Phil.* iv. 4, 5.

I thank Thee, too, that Thou hast made
Joy to abound;
So many gentle thoughts and deeds
Circling us round,
That in the darkest spots on earth
Some love is found.

I thank Thee more that all our joy
Is touched with pain;
That shadows fall on brightest hours,
That thorns remain;
So that earth's bliss may be our guide,
And not our chain.

For Thou, who knowest, Lord, how soon
Our weak heart clings,
Hast given us joys tender and true
But all with wings;
So that we see, gleaming on high,
Diviner things. —*A. Procter.*

And if in this life on earth,
In the chamber, or by the hearth,
Mid the crowded city's tide,
Or high on the lone hillside;
Thou canst make a thought of peace,
Or an aching thought to cease,
Or a gleam of joy to burst
On a soul in sadness nurst,
Spare not thy hand, my child;
Though the gladdened should never know
The well-spring amid the wild,
Whence the waters of blessings flow.

The voice of joy is praise.

Happiness is the temper of holiness, and if the voice of patient anguish is praise to God much more is the clear voice of happiness, a happiness that fastens not on created things, but is centred in Himself. They, whose sunshine is from Him who is within them, worship God brightly out of a blessing which this world cannot touch, because it gushes upwards from a sanctuary which lies too deep for rifling.

Sadness is a sort of spiritual disability. A melancholy man can never be more than a convalescent in the house of God. He may think much of God, but he worships very little.—*Bonaventura.*

As under every stone there is moisture, so under every sorrow there is joy; and when we come to understand life rightly we see that sorrow is after all but the minister of joy; we dig into the bosom of sorrow to find the gold and precious stones of joy.

Sorrow is a condition of time, but joy is the condition of eternity. There are souls in the world which have the gift of finding joy everywhere and of leaving it behind when they go. There is something in their very presence, in their mere silent company, from which joy cannot be extricated or laid aside. It seems as if the shadow of God's own gifts had pressed upon them. They give light without meaning to shine. . . . Who has not known such souls? Who has not owed all that is best in him, after grace, to such as those?—*Faber.*

Take joy home
And make a place in thy great heart for her,
And give her time to grow, and cherish her!
Then will she come and often sing to thee
When thou art working in the furrows; ay,
Or weeding in the sacred hour of dawn.
It is a comely fashion to be glad;
Joy is the grace we say to God. —*J. Ingelow.*

### Travel Notes.

By Our Travelling Correspondent.

### XXII.—AN EASY TRIP FOR NURSES (*continued*).

### Le Mont St. Michel.

As I told you last week, it is quite possible to visit the Mount from St. Servan; but in that way one must miss a good deal, among others, a thorough sight of the curious old town of Dol. I should advise your going there from Saturday to Monday at least, and the most economical way would be to leave St. Servan on Saturday quite early, spend from that day to Monday on the Mount, and return from there to St. Malo in time to take the English boat, which leaves in the evenings—Mondays, Wednesdays, and Saturdays. I say leave St. Servan on Saturday advisedly, because it is important to see Dol on a market day.

### Market Day at Dol.

This is a most curious and unique sight. The market is held in the middle of the principal street, which is lined with sixteenth and even fifteenth century houses. Pigs form the principal merchandise apparently, and the squealing and shrieking is deafening, but the whole thing is most amusing and typical. You must, however, tear yourself from the enthralling scene and visit the grand but rather gloomy cathedral dedicated to St. Sampson. It is sadly in need of repair, especially about the beautiful south porch.

Le Mont St. Michel.

### Arrival at the Mount.

You will have taken your tickets at St. Malo "en correspondance," which insures you a seat on one of the many breaks and vehicles of all kinds waiting at Pontorson to convey you to your destination. To my selfish mind the place is quite spoilt since it has become so tourist-ridden. I knew it in my childish days, and it was then much more impressive in its gloomy isolation, inhabited, as far as the abbey itself was concerned, by about a dozen priests. However, one must admit the pleasure it gives now to many thousands, and the restoration, which was begun by Viollet-le-Duc, is certainly being carried out with great taste and wisdom, though I preferred it in its untouched decay. When you reach the walls you will be besieged by hotel touts. Sternly refuse all but those belonging to Poulard Ainé. The Ainé, remember, is most important, as there is a rival establishment of the same name. I have put up some fifteen times at Poulard Ainé's, and find Madame more fascinating each time. The little sea-girt town is entered by a series of well-defended gates, of which I send you an illustration, with Monsieur le Curé in the foreground, and the gallant military appropriately filling up the portcullis opening. Just the other side of the gateway where the officers are standing is the commencement of the village street. By devious windings you gradually ascend to the principal entrance of the abbey, or, by turning sharply to the right, and mounting what are called the Charles the Seventh steps, you emerge upon the ramparts, which in time also bring you to the "Merveille," that part of the vast conventual pile which you enter first. In olden days I was allowed to wander at will amid the wonders of this huge stone pile; but, alas! now you are solemnly conducted round by a "gardien" at the price of 50 centimes—this latter is, however, not compulsory. The Montgommeries, so called from an incident in the wars of the League, consist of two huge halls with massive pillars. Here was the pulley by which the monks drew up their water from the sacred well of St. Aubert, and by this a treacherous entrance was effected by the Protestant general in 1591.

### The Salle des Chevaliers.

This exquisite hall is situated above the Montgommeries, and is a dream of beauty. Here Louis XI. founded the Order of St. Michael, and here the knights held their chapters and surrounded themselves with the gorgeous insignia of their order. Opening from this lordly room with its hooded fireplaces is the refectory, the roof of which is supported by slender columns.

### The Cloisters.

What can I say adequately of these? They are among the most perfect I have ever seen, though the effect is marred by the gaudy tiling to the roof. I am told it is an exact reproduction of the old work, only lacking the softening effects of time and weather.

From here one passes to the Dortoir, which was terribly mutilated at the time of the great Revolution. It has now been restored, and with great judgment and care.

### The Dungeons.

The various dungeons—dens indeed one might truly call them—cages, and oubliettes are the most popular of all sights on the Mount, and truly there is a weird fascination about them that seizes hold of all. The cages no longer exist, but one space is left that once contained such an instrument of torture, and one can realise the impossibility of any posture but that of crouching for the unfortunate inmate. Then there are the awful twins, two noisome dungeons close together without air and almost without light. A terrible tragedy occurred in one of them about forty years since when the abbey was used as a prison. A prisoner murdered a warder there by dashing out his brains against the wall. Even now, as the work of restoration progresses, from time to time the workmen come upon unknown "oubliettes," or secret prisons, and the discovery of bones show how the unfortunate prisoners were left to starve to death.

The Basilica Itself.

When I knew the Mount first service was held in this noble abbey, but for some years now its use has been abandoned and the little village church suffices for all the religious needs of the Montois. In those days it was very impressive to attend Mass in the early morning, and quaint to be told in a discreet whisper by the priest that there was an unavoidable delay because he was waiting "for a family from the mainland." This was caused by the inconvenience of having to wait for tides before the causeway was constructed.

The Quicksands.

These terrifying natural protectors add a weird interest to the Mount, and fearful are the stories told of the gruesome deaths met with in their depths. Only a few of the inhabitants thoroughly understand their secrets, and it is certain death to venture on the sands without a guide. Within the last few years Pierre le Sauveteur died on the Mount; as his name hints, he had saved many lives, and so perchance redeemed a life that had been wild, ungoverned, and stubborn. Many people regarded him with undisguised fear, and would not have dared to refuse his demands; for indeed Pierre did not scruple to beg. But I must here record that I have spent hours on the sands with Pierre; he never frightened me, and never asked for a sou. He had a dark and romantic history, which some day perhaps I may tell.

### TRAVEL NOTES AND QUERIES.

Rules in Regard to Correspondence for this Section.—All questioners must use a pseudonym for publication, but the communication must also bear the writer's own name and address as well, which will be regarded as confidential. All such communications to be addressed "Travel Editor, 'Nursing Mirror,' 28, Southampton Street, Strand." No charge will be made for inserting and answering questions in the inquiry column, and all will be answered in rotation as space permits. If an answer by letter is required, a stamped and addressed envelope must be enclosed, together with 2s. 6d., which fee will be devoted to the objects of the "Hospital Convalescent Fund." Any inquiries reaching the office after Monday cannot be answered in "The Mirror" of the current week.

St. Servan for a Fortnight (St. Malo).—Write to Madame Pallot, Maison Mazzias, and ask her if she will have rooms for you at seven francs per day pension. You might also try Miss Humphreys, Rue de Pomellec, the same terms; there is a third pension, kept by Miss Dixon, Place Constantine. At Paramé the Hôtel Continental will, I believe, take you for six francs if you write and arrange; also the Hôtel des Bains, but tips will come to rather more than at a pension. You will be able to be abroad a fortnight, and even visit Mount St. Michel (article appearing this week) very comfortably for £8 or £9.

Lower Brittany (Artistic).—Quimper, Quimperlé, and Douarnenez are good stopping places. Yes, it is still cheap, though not so markedly so as 10 years ago. Pont Aven is a great centre for artists. Hotels, Voyageurs and Lion d'Or. Terms very reasonable on arrangement. If you want to study magnificent and gloomy coast scenery be sure to go to Audierne, and from thence to the Pointe du Raz and Baie des Trépassés.

Florence (Between Seasons).—An excellent place for your purpose. From April to the middle of June it is delightful; after that it is too hot for most people. If you wish to stay a little later in the Italian towns, leave Florence at the end of May and go to Venice for three weeks. You will not be oppressed with the heat there. By the end of June you will be able to penetrate the Higher Alps.

Sicily (Night).—The journey is necessarily expensive, the distance covered being so great—first-class, £11 17s. 3d.; second-class, £8 5s. 6d. This is straight through from London to Naples, and thence by steamer. No, there is no fear of brigands if you keep in the well-known tracks.

Italian Lakes (Volunteer).—In July it would be oppressively hot. May and June are the months for the lakes. A personally-conducted party starts on very reasonable terms for the Italian Lakes on May 27th.

*(For Travel Advertisements see Page xx.)*

### To Nurses.

In order to increase and vary the interest in the *Mirror*, we invite contributions from any of our readers in the form of either an article, a paragraph, or information, and will pay a minimum of 5s. for each contribuion. All payments are made at the beginning of each quarter, *i.e.*, January 1st, April 1st, July 1st, and October 1st.

### Notes and Queries.

The contents of the Editor's Letter-box have now reached such unwieldy proportions that it has become necessary to establish a hard and fast rule regarding Answers to Correspondents. In future, all questions requiring replies will continue to be answered in this column without any fee. If an answer is required by letter, a fee of half-a-crown must be enclosed with the note containing the enquiry. We are always pleased to help our numerous correspondents to the fullest extent, and we can trust them to sympathise in the overwhelming amount of writing which makes the new rules a necessity.

Every communication must be accompanied by the writer's name and address, otherwise it will receive no attention.

*Oatmeal and Lanoline Ointment.*

(62) Where can I get an ointment of oatmeal and lanoline? I have lost the address of the chemist from whom I had it.

We do not know of a lanoline and oatmeal ointment, but Burroughs and Wellcome would be the firm to apply to on the subject. They make up many lanoline preparations.

*Consulting Fees.*

(63) I am a reader of The Hospital, and should be obliged if some one would kindly say in a future number whether a doctor ought to ask a fee for merely answering a question regarding the climate of a certain place. A lady one day wrote to a doctor whom she had met once or twice, and who had been his patient for a short time once, and who knew her people, asking him to be good enough to tell her what he thought of the climate in which he resided. But, before answering the question, he said of course she would recognise the fact that he could not answer a question without the customary fee.—*C. A. G.*

A professional man is entitled to his fee for answering any query either verbally or by letter. The lady in question wanted a medical man's opinion as to the suitability of the climate of a certain neighbourhood to her constitution. Why should she presume upon a slight acquaintanceship to obtain knowledge valuable to her without paying market price for it. He acted generously under the circumstances in not giving the advice and charging his fee without warning her what to expect.

*Swollen Glands.*

(64) I suffer from swollen neck glands; would they disqualify me from entering as a probationer in a small hospital or nursing home? They do not affect my general health, and otherwise I am perfectly sound.—*H. P.*

This is certainly a question which only a medical man can answer. Can you not consult your family doctor?

*Nursery Lotion.*

(65) Can you give me a little advice? My little girl, eight years old, attends a middle-class school, where the children are supposed to be respectable, and one would think clean; but in spite of constant washing and searching her head seems to be always infested with vermin. I do not want to cut her hair off (she has a beautiful lot), but I shall have to unless I can discover some preparation to get rid of them. Can you suggest a remedy?—*Mother.*

Most good chemists keep a preparation for the purpose which they call "nursery lotion." They guarantee it to be perfectly harmless to the hair and to the head, whilst it effectually gets rid of the unwelcome visitors.

*Provincial Orthopædic Hospitals.*

(66) I should be much obliged if you could tell me of any special provincial hospital where deformities of the feet, such as club foot, are treated; also of any small manual treating of the same subject.—*M. A. B.*

You will find the names of provincial orthopædic hospitals in "Burdett's Hospitals and Charities," but any general large hospital would have an orthopædic department which would suit your purpose.

*Malarial Fever.*

(67) I would be much obliged to any nurse who would kindly give their experience on English malarial fever. I have been suffering from it for several months, and do not get any better. I have been compelled to give up work, and have been taking frequent changes, but up to the present have not benefited much. I should like to hear if there is any cure for it.—*Nurse G.*

*Furnishing.*

(68) My committee are having a new block added to our infirmary. Will you kindly tell me if there is any book published which will help me in drawing up a list for the furnishing?—*Superintendent.*

We do not know of any book which would be of material use to you, for the furnishing of each hospital and infirmary must be regulated by its own special needs and requirements. The most satisfactory way is to visit one or two modern institutions and note for yourself what is excellent and desirable for your own. If you are in London, a visit to one of the large infirmaries—Chelsea or St. Pancras, for instance—would be most helpful, or nearer home there is the Portsea Island Infirmary at Portsmouth, where also you might go to see the new wards at the Royal Portsmouth Hospital.

*Royal National Pension Fund.*

(69) Please tell me where to apply for particulars of the Nurses' Pension Fund?—*Nurse W.*

Write to the Secretary, Royal National Pension Fund for Nurses, 28, Finsbury Pavement, E.C., who will send you full particulars and advise you.

*Daily Nursing.*

(70) Where can I obtain nursing work during the day, or part day?—*E. C. P.*

Advertise in our columns, and watch for advertisements appearing therein.

### Wants and Workers.

A District Nurse asks: How can I get orders for macramé work for a poor girl, a cripple, who is very clever at it, and wants a market for her work? Can any of our readers help?

## Figures and Tables

**Figure f1:**